# An exploratory phenome wide association study linking asthma and liver disease genetic variants to electronic health records from the Estonian Biobank

**DOI:** 10.1371/journal.pone.0215026

**Published:** 2019-04-12

**Authors:** Glen James, Sulev Reisberg, Kaido Lepik, Nicholas Galwey, Paul Avillach, Liis Kolberg, Reedik Mägi, Tõnu Esko, Myriam Alexander, Dawn Waterworth, A. Katrina Loomis, Jaak Vilo

**Affiliations:** 1 AstraZeneca, Global Medical Affairs, Cambridge, United Kingdom; 2 Institute of Computer Science, University of Tartu, Tartu, Estonia; 3 STACC, Tartu, Estonia; 4 Quretec, Tartu, Estonia; 5 GlaxoSmithKline, Research and Development, Stevenage, United Kingdom; 6 Department of Biomedical Informatics, Harvard Medical School, Boston, United States of America; 7 Department of Medical Informatics, Erasmus University Medical Center Rotterdam, Rotterdam, Netherlands; 8 Estonian Genome Center, Institute of Genomics, University of Tartu, Tartu, Estonia; 9 GlaxoSmithKline, Genetics, Collegeville, PA, United States of America; 10 Pfizer Worldwide Research and Development, Groton, CT, United States of America; Banaras Hindu University, INDIA

## Abstract

The Estonian Biobank, governed by the Institute of Genomics at the University of Tartu (Biobank), has stored genetic material/DNA and continuously collected data since 2002 on a total of 52,274 individuals representing ~5% of the Estonian adult population and is increasing. To explore the utility of data available in the Biobank, we conducted a phenome-wide association study (PheWAS) in two areas of interest to healthcare researchers; asthma and liver disease. We used 11 asthma and 13 liver disease-associated single nucleotide polymorphisms (SNPs), identified from published genome-wide association studies, to test our ability to detect established associations. We confirmed 2 asthma and 5 liver disease associated variants at nominal significance and directionally consistent with published results. We found 2 associations that were opposite to what was published before (rs4374383:AA increases risk of NASH/NAFLD, rs11597086 increases ALT level). Three SNP-diagnosis pairs passed the phenome-wide significance threshold: rs9273349 and E06 (thyroiditis, p = 5.50x10^-8^); rs9273349 and E10 (type-1 diabetes, p = 2.60x10^-7^); and rs2281135 and K76 (non-alcoholic liver diseases, including NAFLD, p = 4.10x10^-7^). We have validated our approach and confirmed the quality of the data for these conditions. Importantly, we demonstrate that the extensive amount of genetic and medical information from the Estonian Biobank can be successfully utilized for scientific research.

## 2. Introduction

Genetic data are an important resource for scientific research and potential drug target identification [1] and genome-wide association studies (GWAS) have identified many disease-associated genetic variants [2]. Complementary to GWAS are phenome-wide association studies (PheWAS) which use a genotype-to-phenotype approach, testing for associations between specific genetic variants over a wide spectrum of phenotypes [3]. Combining genetic data with phenotypes defined and validated in electronic health records (EHRs) permits associations between genetic variants and disease outcomes, including diagnoses and procedures not commonly found in GWAS studies. To date, despite a relative abundance of EHR and genetic data becoming available, few large scale PheWAS studies have linked these data [4]. Understanding the full range of associations along with understanding the functional mechanisms of causal genetic variants will have important implications for the design of novel therapies across indications to reduce morbidity and mortality.

The Estonian Biobank, governed by the Institute of Genomics at the University of Tartu (Biobank) and launched in 2007, aimed to create a biobank with biological samples and genetic material with linkage to EHRs to investigate the genetic, environmental and behavioral background of common diseases in the Estonian population [5]. To explore the utility of data in the Biobank, we conducted a PheWAS in two areas of interest to healthcare researchers–asthma and liver disease–to determine whether established and novel disease associations together with early disease indicators could be detected.

Liver disease is a heterogeneous and complex disease, being the tenth highest cause of mortality worldwide [6]. Non-alcoholic fatty liver disease (NAFLD) is a type of liver disease which is defined by the presence of liver fat accumulation exceeding 5% of hepatocytes, in the absence of significant alcohol intake, viral infection, or any specific aetiology of liver disease. Patients with NAFLD may develop more serious conditions, such as non-alcoholic steatohepatitis (NASH), hepatic fibrosis, cirrhosis, and hepatocellular carcinoma, and there are currently no therapies approved to treat NAFLD or NASH. Due to the surge in prevalence of obesity, NAFLD is now considered more common than alcoholic liver disease. The prevalence of NAFLD, in the general population, is estimated to range between 12–18% in Europe and 27–38% in the US [7]. Single nucleotide polymorphisms (SNPs) associated with obesity, in particular in the Patatin-like Phospholipase 3 (*PNPLA3*) gene, have been associated with liver disease and cirrhosis in GWAS [8–11]. Other SNPs reported to be associated with NAFLD, NASH, liver fat or fibrosis are in the *APOC3*, *GCKR*, *MBOAT7*, *MERTK*, *PPP1R3B*, *SOD2*, *TM6SF2* and *TRIB1* genes [7,8,11–29].

Asthma is a chronic inflammatory disease affecting the airways and lung characterized by recurring symptoms including breathlessness and wheezing caused by inhalation of environmental particulates such as allergens. Asthma requires both acute and long-term management for alleviation of symptoms which vary in severity between individuals. It is estimated that 235 million people worldwide suffer from asthma [30]. A number of asthma associated SNPs have been identified by GWAS and include variants near *TSLP*, *BIRC3*, *IL18R1*, *HLA-DQB1*, *IL33*, *GSDMB*, *GSDM1*, *IL2RB*, *SLC22A5*, *IL13*, and *RORA* gene regions [31–33].

## 3. Objectives

In an effort to assess the utility of the genetic and phenotypic data available in the Estonian Biobank, our first two objectives were to show our ability to detect previously reported GWAS associations of (1) asthma-associated SNPs with biomarkers (lab measures) and clinical diagnoses of asthma, and (2) liver disease-associated SNPs and clinical diagnoses of NAFLD/NASH within the Estonian Biobank. Both objectives are first, to test the Biobank suitability for validation/replication studies, and second, to confirm no systematic errors in the Biobank data before moving on to objective three, to conduct a PheWAS to test the association of the selected 24 SNPs with all other ICD-10 clinical diagnoses. Where significant associations between SNPs and ICD-10 diagnostic codes are found, our fourth objective was to determine associations of corresponding SNPs with lab/biomarker measures (quantitative traits) prior to the date of diagnosis.

## 4. Materials and methods

### Database

The Estonian Biobank has had continuous data collection since 2002 on a total of 52,274 voluntary adult individuals, of which 50,000 are currently active (alive and data continuously updated) in the database, representing approximately 5% of the Estonian adult population. The Biobank has the ability to access Estonian primary care and secondary/inpatient care EHRs via linkage to a central e-health database where EHR information is sent and uploaded by healthcare professionals. Additionally, participant information is updated through linkage to other databases/registries including the Estonian population registry, cancer registry, and death registry. This linkage ensures prospective and retrospective capture of patient and disease characteristics in the form of ICD-10 codes [5], demographics, lifestyle information, laboratory measurements, biomarker measurements, diagnoses and drug utilization (prescriptions and fill data). Additional information is captured by questionnaires, including lifestyle data e.g. smoking and alcohol consumption. Questionnaire information is usually captured during patient enrolment; however, where new questionnaires are introduced, existing Biobank participants are invited to complete these to increase completeness of data. Number of participants in the Biobank with genetic data available is expected to increase over 150K by the end of 2019.

### Case ascertainment

To be eligible for the study, patients were required to have genetic and EHR data linkage for at least one year (365 days) after recruitment. Patients were excluded if age, gender or genetic information was missing or if patients had no EHR linkage. ICD-10 diagnostic codes were used to determine clinical diagnoses.

### SNP selection and array information

We conducted a thorough search of the literature to identify and select genetic variants associated with asthma or fatty liver disease (or hepatic fat) using GWAS methodology [7–21,23–29,31–38]. For one variant (rs4240624), the published association was given with computed tomography measured hepatic steatosis [25] and we wanted to test whether we can identify the association with NAFLD also. We then determined the availability of these SNPs on the Illumina Infinium Global Screening Array used by the Biobank to obtain genetic information. Where primary SNPs of interest were not available on this array, the Broad Institute SNP Annotation and Proxy Search (SNAP) tool was used to identify proxy SNPs (SNPs which can represent the primary SNP of interest) with a minimum linkage disequilibrium r^2^≥0.6 [39]. After these steps, 11 asthma (1 proxy) and 13 liver disease-associated (8 proxies) SNPs of interest were included in our analyses (**Table 1**). For 4 of these SNPs, associations with one or more lab measurements (6 in total) were reported.

**Table 1:** SNPs / Proxy SNPs of the genes of interest.

Gene	Phenotype	SNP	Proxy SNP*	R^2^*	Effect Allele (%)	Hardy-Weinberg Equilibrium p-value
**Asthma Phenotype**						
*TSLP*	Asthma, increased eosinophil count, decreased neutrophil count	rs1837253	NA	NA	C (71)	0.190
*BIRC3*	Asthma, reduced eosinophil and neutrophil count	rs7127583	rs2846848	0.677	T (29)	0.011
*IL18R1*	Asthma	rs3771166	NA	NA	A (26)	0.374
*HLA-DQB1*	Asthma	rs9273349	NA	NA	C (57)	0.316
*IL33*	Asthma	rs1342326	NA	NA	C (10)	0.194
*GSDMB*	Asthma	rs2305480	NA	NA	A (43)	0.261
*GSDM1*	Asthma	rs3894194	NA	NA	A (46)	0.210
*IL2RB*	Asthma	rs2284033	NA	NA	A (46)	0.994
*SLC22A5*	Asthma	rs2073643	NA	NA	C (48)	0.567
*IL13*	Asthma	rs1295686	NA	NA	C (69)	0.751
*RORA*	Asthma	rs11071559	NA	NA	T (19)	0.684
**Liver Phenotype**						
*APOC3*	Hepatic Fat	rs2854117	rs2849176	0.714	T (42)	0.948
*APOC3*	Hepatic Fat	rs2854116	NA	NA	C (44)	0.939
*GCKR*	NAFLD	rs780094	NA	NA	T (39)	0.306
*GCKR*	NAFLD	rs1260326	rs780094	0.933		
*MBOAT7*	NAFLD	rs641738	NA	NA	T (42)	0.239
*MERTK*	HCV fibrosis progression, NAFLD fibrosis^φ^	rs4374383	NA	NA	A (36)	0.347
*PNPLA3*	NAFLD / NASH	rs738409	rs2281135	0.688	A (19)	0.362
*PNPLA3*	Liver density, NAFLD	rs2294918	rs8418	0.890	G (60)	0.388
*PPP1R3B*	Computed tomography measured hepatic steatosis	rs4240624	rs4841132	1.000	G (91)	0.835
*SOD2*	Fibrosis in NAFLD	rs4880	NA	NA	G (55)	0.021
*TM6SF2*	NAFLD/NASH	rs58542926	NA	NA	T (7)	0.721
*TRIB1*	NAFLD, Lipids	rs2954021	rs2980875	0.780	A (47)	0.959
*HSD17B13*	NAFLD, increased ALT level	rs6834314	rs9992651	0.823	G (77)	0.338
*ERLIN1*	NAFLD, decreased ALT level	rs2862954	rs11597086	0.669	C (41)	0.878

*Where primary SNPs of interest were not available on this array, the Broad Institute SNP Annotation and Proxy Search (SNAP) tool was used to identify appropriate proxy SNPs (SNPs which can represent the SNP of interest) with a minimum r^2^ value of 0.6. r^2^ or linkage disequilibrium is the non-random measure of association between alleles at different loci, providing an approximate reliability for a proxy SNP representing a primary SNP.

### PheWAS codes

ICD-10 diagnostic codes were mapped to “PheWAS codes” using methodology from Neuraz et al. [40]. This involved converting individual ICD-10 codes into a higher order of grouped codes for a single disease, e.g. grouping codes A00.0, A00.9, A00.1, A00 for cholera into a three-character code A00 (“PheWAS code”) and using broader A00-A09 range (intestinal infectious diseases) as an exclusion criterion for the control group (S1 Table). If a significant association was observed, we repeated the analysis using the individual ICD-10 code to define the case group while keeping the control group criteria unchanged, *e*.*g* A00.0 as the case group and A00-A09 as exclusion criteria.

### Other covariates, laboratory measures and biomarkers

To describe the patient population, we extracted data on a range of covariates including birth year, gender, ethnicity, smoking status and body mass index (BMI). Laboratory and biomarker measures included sodium, potassium, blood urea, blood glucose, Creatinine, c-reactive protein, haemoglobin, platelet count, B-type natriuretic peptide / brain natriuretic protein, troponin I, troponin T, white blood cell count, neutrophil count, eosinophil count, serum uric acid, fibrinogen, A-fetoprotein, gamma-glutamyl transferase, alanine transaminase (ALT), alkaline phosphatase, albumin, aspartate transaminase, bilirubin, total cholesterol, high-density lipoprotein cholesterol and low density lipoprotein cholesterol.

### Data extraction and analysis

Biobank participant information, e.g. lifestyle information, was extracted directly from the Estonian Biobank questionnaires completed during recruitment. Incident diagnoses were extracted from local copy of central e-health database, and laboratory measures were extracted using both e-health database and local copies of two main Estonian hospitals’ databases. In total, there were more than 1.6 million diagnoses recorded of 12,845 different ICD-10 codes, which were grouped into 1,888 higher level ICD-10 (PheWAS) codes used in this analysis. There were over 640,000 lab/biomarker measurements available; however, for 42% of the participants no measurements were recorded. Summary statistics for each laboratory measurement can be found in S1 Text.

SNP data was called from the Illumina Infinium Global Screening chip array. After variant calling, the data was filtered using PLINK software [41] sample-wise: call rate>95%, no sex mismatches between phenotype and genotype data, heterozygosity within 3 standard deviations of average heterozygosity over all samples to eliminate possible inbreeding and DNA contamination; and marker-wise: HWE p-value>1x10^-6^, callrate>95%, and Illumina GenomeStudio GenTrain score >0.6, Cluster Separation Score >0.4. SNPs of interest were then extracted from the data. Analysis of this study was conducted in R version 3.4.3 using the PheWAS R package [42] customized to allow use of ICD-10 codes. The high-performance computing center at the University of Tartu was used for analysis. Calculations for statistical power can be found in S2 Text.

Logistic regression models were used to evaluate association of SNP/proxy SNP variation with ICD-10 diagnoses. Odds ratios (ORs) and 95% confidence intervals (CI) were estimated. Linear regression models were used to evaluate associations between SNPs and laboratory measurements. Most of the laboratory measurements (15 out of 26) were log-transformed due to positive skewness assessed graphically (see Fig A in S1 Text). Prior to these log-transformations, zero values were replaced with the minimum non-zero value in the same variable. Data for laboratory measures were taken from 2008 to 2013 (Fig A in S3 Text) as the central collection of lab measurements had only just started and not all labs recorded the lab test results until 2008, possibly causing a bias if we were to include the earlier measurements in the analysis.

For objective 1, cases for asthma were defined as ever having been diagnosed with ICD-10 codes J45-J46, and controls were defined from the remaining individuals as never (prospectively and retrospectively in the patients’ medical records) having been diagnosed with ICD-10 codes J40-J47. Similarly, for objective 2, cases for NASH/NAFLD were defined as having ICD-10 codes K75.8 or K76.0 and controls as individuals never having been diagnosed with ICD-10 codes K70-K77. Full list of J40-J47 and K70-K77 diagnoses with their counts is given in S4 Text. Altogether, 21 previously reported SNP-disease and 6 SNP-lab measurement (covering 4 SNPs out of 24) association validation tests were performed in the afore-mentioned objectives. For objective 3 (PheWAS), each PheWAS code was tested individually, and only PheWAS codes with at least 20 cases and 20 controls were included in the analyses. To explore what drives the detected significant associations, an exact ICD-10 code instead of PheWAS code was used in the subsequent analysis.

A p-value significance threshold 0.05 was used for objectives 1 and 2 (confirmation of liver and asthma SNP associations). For objective 3, a p-value of 2.0x10^-6^ (≈0.05/1,000/24 where 1,000 is the effective number of ICD-10 diagnoses tested and 24 is the number of SNPs tested) was used to reduce the likelihood of chance associations and identify the most prominent differences. To search for evidence of systematic bias, a QQ-plot of PheWAS p-values was used to evaluate whether the observed distribution was different from what would be expected under the null hypothesis.

### Protection of human subjects

Research at Estonian Biobank is regulated by Human Gene Research Act and all participants have signed a broad informed consent. IRB approval for current study was granted by Research Ethics Committee of University of Tartu, approval nr 236/T-23.

## 5. Results

After applying the exclusion criteria, 26,766 (51.2%) Caucasian individuals remained from the original 52,274 (**Table 2**). The main reason for the drop in the number of samples is missing genotype data (not genotyped with the given genotyping array). In this study, most participants were female (71.8%), 41.6% of individuals had a body mass index (BMI) of between 18.5–25 (normal weight), 59.2% were never smokers and 75.9% were of Estonian nationality (**Table 3**).

**Table 2:** Biobank study attrition.

Exclusion Criteria Applied	Number of Patients Remaining (%)	Number of Patients Removed
Total Database Population	52,274 (100)	NA
Has Genotype Data	32,831 (62.8)	19,443
Has EHR Linked Data	26,808 (51.3)	6,023
Inside Study Period	26,789 (51.2)	19
Age >18	26,766 (51.2)	23
Not Missing Gender	26,766 (51.2)	0

**Table 3:** PheWAS study participant characteristics.

Characteristic	N	%
Gender		
Female	19,224	71.8
Male	7,542	28.2
Smoking Status		
Current	7,151	26.7
Former	3,721	13.9
Never	15,853	59.2
Unknown	41	0.2
Body Mass Index		
<18.5	467	1.7
18.5–25	11,127	41.6
25–30	8,721	32.6
30+	6,423	24.0
Unknown	28	0.1
Nationality		
Estonian	20,320	75.9
Russian	5,307	19.8
Other	1,139	4.3

### Preselected SNPs and risk of asthma and fatty liver disease

Two asthma-associated SNPs (rs11071559 (*RORA*) and rs1837253 (*TSLP*)) passed the significance threshold of <0.05 (**Table 4**) and were directionally consistent with previous studies. While Moffat *et al*. showed that rs1837253 significantly reduces severe asthma in one of their datasets, it did not replicate in the other and the association direction with asthma was the opposite (increase) in the full dataset [31]. We also observe the increased effect of allele C of rs1837253 in our data. From the lab measurement tests, we confirmed that each additive effect allele T of rs2846848 (*BIRC3*) had a significant effect on reducing neutrophil levels (**Table 4**). All other associations were non-significant.

**Table 4:** Association between genetic variants and asthma (J45-J46) diagnosis/laboratory measurements in the Estonian Biobank.

Gene	SNP / Proxy SNP and effect allele	Author	Study Size	Study Effect: OR (95% CI), p-value	Biobank Case/Control Size (total size for continuous variables)	Biobank Effect Size: OR (95% CI), p-value
*RORA*	rs11071559:T	Moffat [31]	10,365/16,110	OR = 0.85 (0.79–0.90), p = 7.9E-07	3,424/21,020	**OR = 0.88 (0.83–0.95), p = 0.00036**
*TSLP*	rs1837253:C	Moffat [31]	290/974	Severe asthma OR = 0.56 in one dataset (p = 3x10^-6^); Asthma OR = 1.15 (1.08–1.22), p = 7.5x10^-8^	3,424/21,020	**Asthma OR = 1.08 (1.02–1.15), p = 0.0059**
		Astle [33]	Total study size 173,480 (case/control size not reported)	Increased Eosinophil Count (p = 2.1x10^-17^); Decreased Neutrophil Count (p = 3.5 x10^-12^)	8,040 Eosinophil measurements; 8,174 Neutrophil measurements	Eosinophil/neutrophil counts increased/decreased, but non-significant
*IL33*	rs1342326:C	Moffat [31]	10,365/16,110	OR = 1.22 (1.14–1.30), p = 1.4 x10^-8^	3,424/21,020	OR = 1.08 (0.99–1.17), p = 0.07
*BIRC3*	rs7127583 / rs2846848:T	Roscioli [32]	401	Reduced Eosinophil Count p = 0.002; Reduced Neutrophil Count p = 0.005	8,040 Eosinophil measurements; 8,174 Neutrophil measurements	**Reduced Neutrophil Count (p = 0.018)**; Reduced Eosinophil Count globally non-significant
*IL18R1*	rs3771166:A	Moffat [31]	10,365/16,110	OR = 0.87 (0.83–0.91), p = 1.7x10^-8^	3,424/21,020	OR = 0.97 (0.92–1.03), p = 0.34
*HLA-DQB1*	rs9273349:C	Moffat [31]	10,365/16,110	OR = 1.19 (1.13–1.25), p = 2.0x10^-11^	3,424/21,020	OR = 0.99 (0.94–1.04), p = 0.68
*GSDMB*	rs2305480:A	Moffat [31]	10,365/16,110	OR = 0.82 (0.79–0.86), p = 3.3x10^-16^	3,424/21,020	OR = 0.98 (0.93–1.03), p = 0.47
*GSDM1*	rs3894194:A	Moffat [31]	10,365/16,110	OR = 1.18 (1.13–1.23), p = 2.0x10^-13^	3,424/21,020	OR = 1.03 (0.98–1.09), p = 0.25
*IL2RB*	rs2284033:A	Moffat [31]	10,365/16,110	OR = 0.90 (0.86–0.94) p = 4.8x10^-6^	3,424/21,020	OR = 0.98 (0.93–1.03), p = 0.37
*SLC22A5*	rs2073643:C	Moffat [31]	10,365/16,110	OR = 0.90 (0.86–0.94) p = 6.2x10^-6^	3,424/21,020	OR = 0.98 (0.93–1.03), p = 0.35
*IL13*	rs1295686:C	Moffat [31]	10,365/16,110	OR = 0.87 (0.83–0.92), p = 7.9x10^-7^	3,424/21,020	OR = 0.97 (0.92–1.03), p = 0.32

Five fatty liver disease-associated SNPs (rs780094 (*GCKR*), rs2281135 (*PNPLA3*), rs8418 (*PNPLA3*), rs58542926 (*TM6SF2*), rs2980875 (*TRIB1*)) passed the significance threshold of <0.05 (**Table 5**) and were directionally consistent with previous studies. Additionally, we found that the AA genotype of rs4374383 (*MERTK*) significantly increases the chance of developing NASH/NAFLD, which is the opposite direction of effect to what was shown by Patin *et al*. [29]. For rs9992651 (*HSD17B13*) and rs11597086 (*ERLIN1*) we also assessed the association to ALT levels. In our analysis, both SNPs are significantly associated with increased ALT levels, but rs11597086 was in the opposite direction of what has been shown by Yuan *et al*. [21].

**Table 5:** Association between genetic variants and NASH/NAFLD diagnosis (K75.8 or K76.0)/laboratory measurements in the Estonian Biobank.

Gene	SNP / Proxy SNP	Author	Study Size	Study Effect	Biobank Case/Control Size (total size for continuous variables)	Biobank Effect Size: OR (95% CI), p-value
APOC3	rs2854117 / rs2849176:T	Petersen [23]	258, prevalence of NAFLD not reported	Increased prevalence of NAFLD (p<0.001)	625/25,097	OR = 1.00 (0.89–1.12), p = 0.99
APOC3	rs2854116:C					OR = 1.02 (0.91–1.14), p = 0.79
GCKR	rs780094:T	Speliotes [25]	592/1,405	Effect allele T: NAFLD OR = 1.45 (1.17–1.57), p = 2.6x10^-8^		**OR = 1.14 (1.02–1.27),p = 0.026**
		Yang [26]	436/467	Effect allele T: NAFLD OR = 1.61 (1.14–2.27), p = 0.0072		
GCKR	rs1260326:T / rs780094:T	Petit [27]	201/107	Steatosis, OR = 1.99 (1.14–3.47), p = 0.01		
MBOAT7	rs641738:T	Mancina [28]	2,736, case group size not reported	NAFLD OR = 1.20 (1.05–1.37), p = 0.006		OR = 1.03 (0.92–1.16), p = 0.55
MERTK	rs4374383:A	Patin [29]	57/239	Advanced fibrosis OR = 0.18 (0.09–0.36), p = 1.1x10^-9^ (recessive model, AA required)		**OR = 1.12 (1.01–1.26), p = 0.03**
PNPLA3	rs738409:G / rs2281135:A	Kitamoto [24]	540/1,012	NAFLD OR = 2.20 (1.78–2.72), p = 4.1x10^-13^		**OR = 1.40 (1.23–1.59), p = 4.9x10**^**-7**^
		Speliotes [25]	592/1,405	NAFLD OR = 3.26 (2.11–7.21), p = 3.6x10^-43^		
PNPLA3	rs2294918:G / rs8418:G	Donati [12]	142/100	NAFLD, p = 0.0009, OR not given		**OR = 1.14 (1.01–1.28), p = 0.030**
PPP1R3B	rs4240624:A / rs4841132:G	Speliotes [25]	592/1,405	Significant effect for computed tomography measured hepatic steatosis (p = 3.6x10^-18^); NAFLD OR = 0.93 (0.68–1.18), p = 0.285		OR = 0.86 (0.71–1.03), p = 0.10
SOD2	rs4880:G	Al-Serri [16]	179/323	Advanced fibrosis OR = 1.56 (1.09–2.25), p = 0.014		OR = 1.00 (0.90–1.12), p = 0.96
TM6SF2	rs58542926:T	Bale [17]	256/247	NAFLD OR = 2.7 (1.37–5.3), p = 0.0004		**OR = 1.29 (1.06–1.58), p = 0.013**
		Liu [15]	437/637	Advanced fibrosis OR = 1.88 (1.41–2.5), p = 1.6x10^-5^		
TRIB1	rs2954021:A / rs2980875:A	Kitamoto [24]	540/1,012	NAFLD OR = 1.52, (1.23–1.88), p = 9.7x10^-5^		**OR = 1.20 (1.07–1.34), p = 0.001**
HSD17B13	rs6834314/ rs9992651:G	Chambers [43]	61,089	Increase of ALT concentration in plasma per copy of effect allele rs6834314 A: OR = 2.6, (1.9–3.4), p = 3.1x10^-9^	9,107 ALT measurements	**Increased ALT Count (p = 0.012)**
ERLIN1	rs2862954 / rs11597086:C	Yuan [21]	7,715	rs11597086 C: Decreased ALT level (p = 1.8x10^-8^)	9,107 ALT measurements	**Increased ALT level (p = 0.0056)**

**Fig 1:**
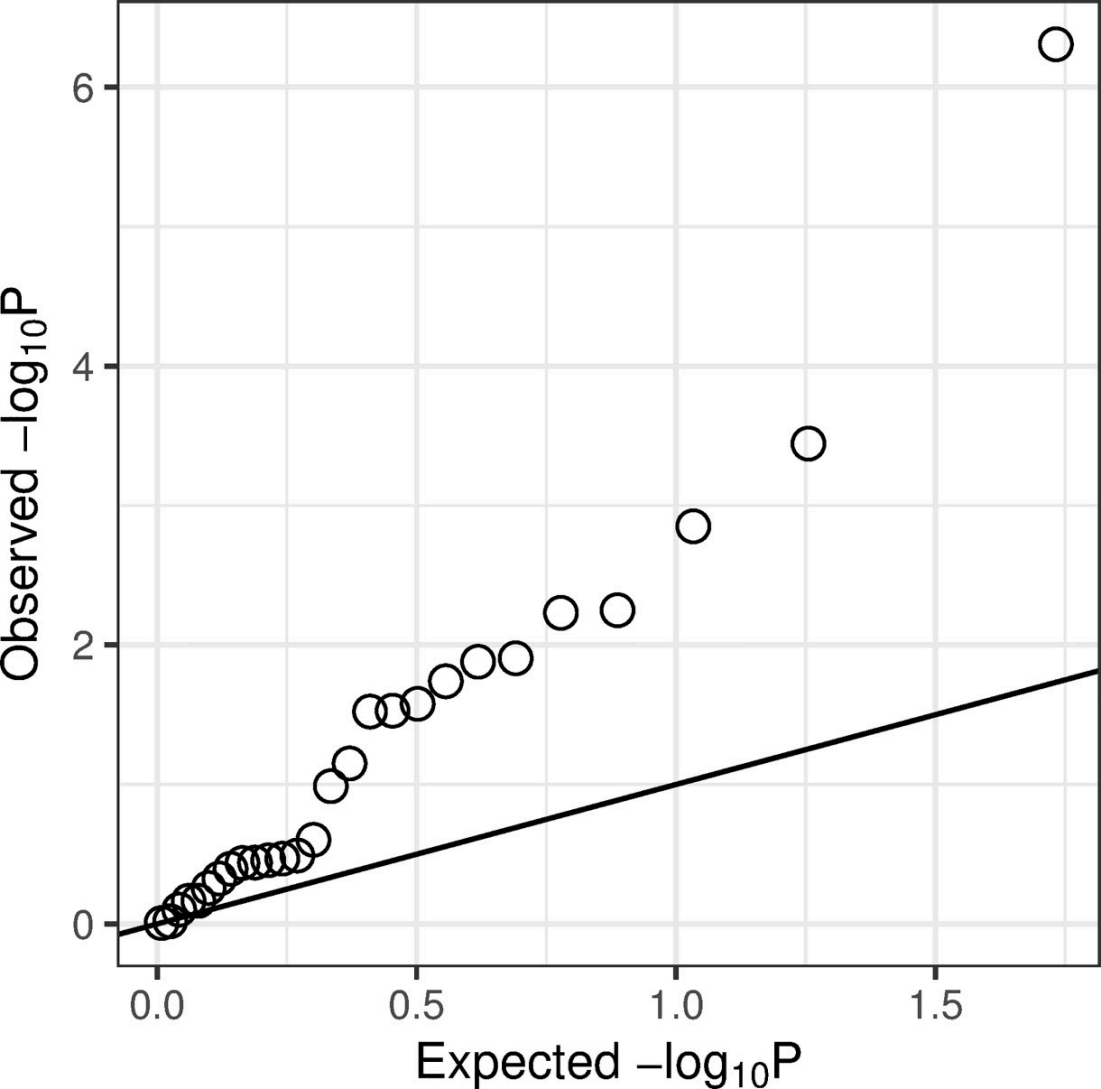
QQ-plot of p-values of the associations between SNPs and asthma/liver disease and lab measures (objectives 1–2).

We could only replicate some previously reported associations with asthma or fatty liver disease-associated SNPs, but this is likely due to having only a small number of cases/measurements for each of these conditions (Tables 4 and 5) and limitations of EHRs and ICD-10 codes which might not always result in clear signals for all diseases (see Discussion). However, while many of the associations we attempted to confirm were not strong enough in our analysis to pass the significance threshold, almost all of the effect directions were concordant between our study and the original studies, and the QQ-plot of p-values from our confirmatory analysis showed clear enrichment of small p-values (**Fig 1**). We can consequently expect the Estonian Biobank data to be suitable for the PheWAS of these SNPs, though we might lack sufficient power to detect modest association signals for specific diseases.

### PheWAS association between pre-selected SNPs and ICD-10 codes

**Fig 2:**
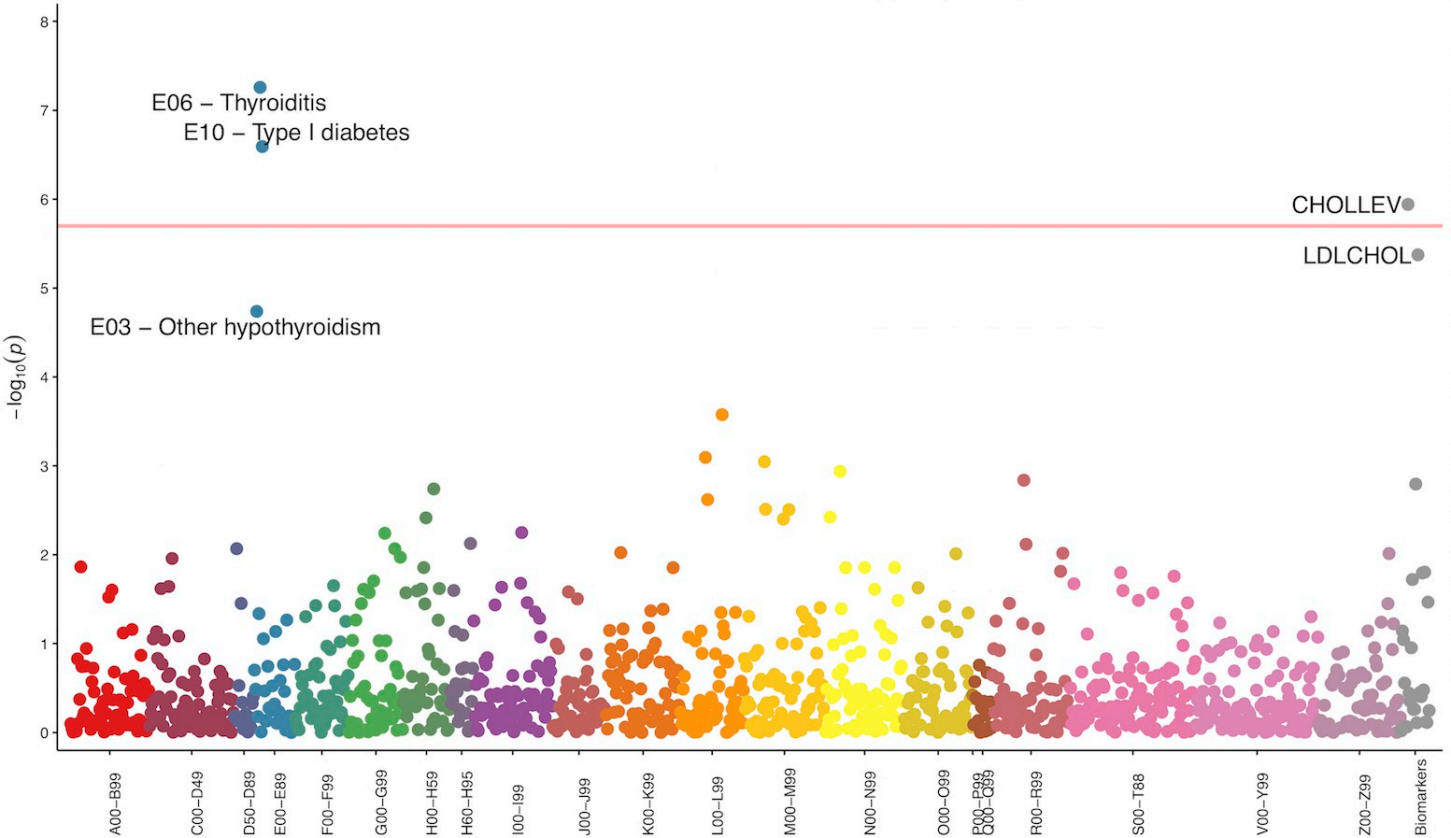
PheWAS Plot for rs9273349. Pink line corresponds to the significance threshold. Groups were defined by the ICD-10.

We conducted a PheWAS to test the association of 24 investigated asthma and liver disease SNPs with all ICD-10 clinical diagnoses. Three SNP-diagnosis pairs passed the PheWAS significance threshold: rs9273349 (*HLA-DQB1)* and E06 (thyroditis); rs9273349 (*HLA-DQB1)* and E10 (type-1 diabetes) **(Table 6, Fig 2)**; and rs2281135 (*PNPLA3*) and K76 (non-alcoholic liver diseases, including NAFLD) (**Table 6**). The QQ-plot of all the association p-values (Fig B in S2 Text) does not indicate any systematic biases in our study (*e*.*g*. due to population stratification) as some inflation is expected due to analyzing known disease-associated SNPs.

**Table 6:** Results of the PheWAS using the PheWAS codes and passing the PheWAS significance threshold.

Phenotype	SNP	Single diagnosis required, Number of cases/controls	Single diagnosis required, OR (95% CI), p-value
E06 (Thyroiditis)	rs9273349	2,458/20,382	OR = 1.182 (1.113–1.255), p = 5.52x10^-8^
E10 (Type 1 Diabetes)	rs9273349	719/23,268	OR = 1.331 (1.194–1.484), p = 2.55x10^-7^
K76 (non-alcoholic liver diseases, including NAFLD)	rs2281135	1,041/25,097	OR = 1.309 (1.179–1.452), p = 4.05x10^-7^

In order to explore what exact diagnoses drive the detected associations, we repeated the analysis with the individual ICD-10 diagnosis codes instead of PheWAS codes (**Table 7**). We found that rs9273349 is associated with autoimmune thyroiditis (E06.3) and insulin-dependent diabetes mellitus without complications (E10.9), with ophthalmic complications (E10.3) and with multiple complications (E10.7). rs2281135 is associated with fatty (change of) liver, not elsewhere classified, including NAFLD (K76.0).

**Table 7:** Results of the PheWAS using exact ICD-10 diagnosis codes and passing the PheWAS significance threshold.

Phenotype	SNP	Single diagnosis required, Number of cases/controls	Single diagnosis required, OR (95% CI), p-value
E10.7 (Insulin-dependent diabetes mellitus with multiple complications)	rs9273349	215/23,268	OR = 2.021 (1.634–2.500), p = 8.6x10^-11^
E06.3 (Autoimmune thyroiditis)	rs9273349	1,986/20,382	OR = 1.203 (1.126–1.286), p = 4.7x10^-8^
E10.9 (Insulin-dependent diabetes mellitus without complications)	rs9273349	288/23,268	OR = 1.630 (1.367–1.943), p = 5.3x10^-8^
E10.3 (Insulin-dependent diabetes mellitus with ophthalmic complications)	rs9273349	106/23,268	OR = 2.352 (1.720–3.216), p = 8.6x10^-8^
K76.0 (Fatty (change of) liver, not elsewhere classified, including NAFLD)	rs2281135	605/25,097	OR = 1.251 (1.251–1.630), p = 1.2x10^-7^

### Associations between significant PheWAS results and laboratory measurements

Using the rs9273349-E06, rs9273349-E10 and rs2281135-K76 pairs, the lab/biomarker measures (quantitative traits) closest in time prior to diagnosis were identified but no significant effects were observed under a significance threshold 9.6x10^-4^ (≈0.05/2/26 where 2 is the number of distinct SNPs and 26 is the number of lab/biomarker measures) due to very small sample sizes after discarding all measurements on patients without the underlying diagnosis. Using all the measurements regardless of whether a patient had been diagnosed with a disease or not, only rs9273349 (*HLA-DQB1)* passed the PheWAS significance threshold for decreasing average (over all patient’s measurements) levels of cholesterol per addition of an effect allele (p = 1.1x10^-6^) (**Fig 2**).

## 6. Discussion

This is the first PheWAS study using Estonian Biobank data linked to EHRs. Large scale PheWAS are scarce [44] with other PheWAS utilising mostly small cohorts [42,45–52]. Recently large scale PheWAS have become possible in the UK Biobank, but other biobanks will still be required for further validation or replication. To assess the Estonian Biobank’s suitability for such a study or association validation/replication purposes, we focused on asthma and liver disease associated SNPs only and as a first task, investigated whether the effects previously reported in the literature (GWAS) could also be detected in our data. That is also to confirm that the data have no systematic errors and are suitable for PheWAS analysis. We replicated previous GWAS results, reporting a significant association of *TSLP* and *RORA* gene variants with asthma [31,33] (**Table 4**) and *GCKR*, *PNPLA3*, *TRIB1* and *TM6SF2* gene variants with the risk of developing liver diseases, notably NAFLD/NASH [8,11–15,20,24–26,29,53] (**Table 5**). We observed decreasing neutrophil levels per addition of an effect allele in the *BIRC3* gene variant, consistent with previously reported results. In addition, variants in *HSD17B13* and *ERLIN1* influence alanine transaminase (ALT) levels. The *HSD17B13* association is consistent with a recent study reporting that a loss-of-function variant associated with decreased levels of ALT and aspartate aminotransferase (AST) and reduced the risk of liver disease and progression from NAFLD to NASH [22]. Our observed association between rs11597086 (*ERLIN1*) and ALT level is in the opposite direction of what has been previously reported [21].

The limited replication of our results with previously published GWAS results in asthma and liver disease is likely due to smaller case sizes in our data than in the original studies, leading to low power, but with continuous enrolment and data collection, statistical power will improve in time or could be enhanced by meta-analysis with other studies.

From the PheWAS, we identified the association of asthma-associated genetic variant rs9273349 with type 1 diabetes, and autoimmune thyroiditis. rs9273349 is a variant of the major histocompatibility complex, class II, DQ beta 1 (*HLA-DQB1*) gene which has an important role in the immune system. *HLA-DQB1* is anchored to the cell surface membrane and functions to present extracellular proteins into the cell [54]. A number of studies have identified the association of *HLA-DQB1* with thyroiditis (notably autoimmune Hashimoto’s thyroiditis) and type 1 diabetes [55–59]. Recently, a study by Verma *et al*. detected an association between acquired hypothyroidism (usually caused by thyroiditis) and rs17843604, a SNP in the same *HLA-DQA1/B1* region [44]. Thyroiditis is a complex immune disorder of unknown aetiology where an infiltration of T and B lymphocytes occurs as a reaction to thyroid antigens. These B and T lymphocytes then produce thyroid autoantibodies resulting in clinical hypo- or hyper-thyroidism [55]. Type 1 diabetes is also a T lymphocyte driven disease which results in the destruction of insulin producing pancreatic islet cells. As suggested in [31,60,61], the amino acid variation of *HLA-DQB1* variants could cause differential and incorrect binding of peptides, altering cellular sensitization resulting in cellular hypo- or hyperactivity, disrupting homeostatic immune function. Additionally, Mosaad *et al*. observed microalbuminuria in type 1 diabetic patients, suggesting that alterations in *HLA-DQB1* expression effect homeostatic regulation [59].

This study has a number of strengths which include; a large sample size of ICD-10 diagnoses and quantitative measures linked to 26,766 genotyped individuals, relatively long follow-up time, integration of questionnaire data and linkage of EHR data with laboratory and other databases. However, there are a number of limitations to consider. The large proportion of females may introduce bias and reduce generalizability to the general population. However, this is a general problem of all voluntary biobank cohorts as women tend to enroll more actively than men [5]. Furthermore, EHR-linked data are not collected for research purposes, and without strict standards for data collection and format, the quality of the data may vary broadly (missing data, non-coherent format, errors on data insertion etc.). Even when an association between a SNP and an ICD-10 diagnostic code or biomarker/lab value is clearly identified, this does not imply a causal variant. ICD-10 coding may be a limitation itself as in some cases it can be difficult to ascertain the exact condition. The limitation of using a biomarker/lab measure closest to diagnosis is that it may not be the most sensitive or specific predictor of disease. Additionally, these measures do not take into account potential confounders e.g. response to medication, age and time-period effects. Furthermore, small sample sizes for specific conditions limit statistical power. For example, many diseases are rare and due to the fine granularity of ICD-10 codes, using the exact codes results in very small sample sizes for case groups. With the small number of cases, the power to detect an association is limited. Therefore, it is helpful to conduct a two-step PheWAS by first using higher level diagnosis codes to screen for any association signals, and subsequently evaluating the more detailed codes to understand where specifically the association is coming from. In our study, after detecting the association between rs9273349 and E06 (thyroiditis), an exact diagnosis analysis revealed that the association was driven by E06.3 (autoimmune thyroiditis). Similarly, the association between rs2281135 and K76 “Other diseases of liver, non-alcoholic fatty liver disease (NAFLD)” is actually driven by more specific code K76.0 “Fatty (change of) liver, not elsewhere classified”.

In conclusion, this is the first PheWAS conducted using the Estonian Biobank demonstrating the extensive amount of genetic and medical information which can be successfully utilized for scientific research. The Estonian Biobank has 50K participants, all have signed informed consent, allowing regular data update from all health databases throughout their lives. Notably, its value is increasing over time—not only because of the continuous EHR data addition and vast amount of genetic data available, but also the participation count of Estonian Biobank (with genetic data available) is expected to rise to 150K by the end of 2019. We showed that Biobank data can be effectively used as a validation/replication database. We replicated 9 GWAS associations for asthma and liver disease-associated SNPs and found the opposite effect directions for 2 associations (rs4374383 increases the risk of NAFLD, rs11597086 increases ALT level). Furthermore, this PheWAS exploring the association of 11 asthma-associated and 13 liver disease-associated SNPs with other diseases and biomarkers is one of few studies to use ICD-10 diagnostic codes to link genetic data with EHRs. Although we did not detect any novel associations in our study, we were able to confirm 3 phenome-wide significant associations based on our data–rs9273349 and thyroiditis, rs9273349 and type-1 diabetes, rs2281135 and non-alcoholic liver diseases, including NAFLD. Considering also the continuous addition of the new data, this highlights the usability of Estonian Biobank for using it effectively as a validation database and conducting extended PheWAS studies with much larger sets of SNPs in the future.

## Supporting information

S1 Table ICD-10 codes to Phewas code map. (XLSX)

S1 Text Summary statistics and distributions for each laboratory/biomarker measurements. (DOCX)

S2 Text Calculations for statistical power. (DOCX)

S3 Text Supplementary figures. (DOCX)

S4 Text Liver disease and Asthma ICD-10 diagnostic codes with occurrence and patient count. (DOCX)

## References

[1] Nelson MR, Tipney H, Painter JL, Shen J, Nicoletti P, Shen Y, et al The support of human genetic evidence for approved drug indications. Nat Genet. 2015;47: 856–860. 10.1038/ng.3314 26121088

[2] Hindorff LA, Sethupathy P, Junkins HA, Ramos EM, Mehta JP, Collins FS, et al Potential etiologic and functional implications of genome-wide association loci for human diseases and traits. Proc Natl Acad Sci. 2009;106: 9362–9367. 10.1073/pnas.0903103106 19474294 PMC2687147

[3] Hebbring SJ. The challenges, advantages and future of phenome-wide association studies. Immunology. 2014 pp. 157–165. 10.1111/imm.12195 24147732 PMC3904236

[4] Kerr SM, Campbell A, Marten J, Vitart V, McIntosh AM, Porteous DJ, et al Electronic health record and genome-wide genetic data in Generation Scotland participants. Wellcome Open Res. 2017;2: 85 10.12688/wellcomeopenres.12600.1 29062915 PMC5645708

[5] Leitsalu L, Haller T, Esko T, Tammesoo ML, Alavere H, Snieder H, et al Cohort profile: Estonian biobank of the Estonian genome center, university of Tartu. Int J Epidemiol. 2015;44: 1137–1147. 10.1093/ije/dyt268 24518929

[6] Institute for Health Metrics and Evaluation. Global Burden of Disease Study 2016 (GBD 2016) Results In: Global Burden of Disease Collaborative Network 2017.

[7] Anstee QM, Day CP. The genetics of NAFLD. Nature Reviews Gastroenterology and Hepatology. 2013 pp. 645–655. 10.1038/nrgastro.2013.182 24061205

[8] Tian C, Stokowski RP, Kershenobich D, Ballinger DG, Hinds DA. Variant in PNPLA3 is associated with alcoholic liver disease. Nat Genet. 2010;42: 21–23. 10.1038/ng.488 19946271

[9] Santoro N, Kursawe R, D’Adamo E, Dykas DJ, Zhang CK, Bale AE, et al A common variant in the patatin-like phospholipase 3 gene (PNPLA3) is associated with fatty liver disease in obese children and adolescents. Hepatology. 2010;52: 1281–1290. 10.1002/hep.23832 20803499 PMC3221304

[10] Dongiovanni P, Anstee QM, Valenti L. Genetic predisposition in NAFLD and NASH: impact on severity of liver disease and response to treatment. Curr Pharm Des. 2013;19: 5219–38. 10.2174/13816128113199990381 23394097 PMC3850262

[11] Romeo S, Kozlitina J, Xing C, Pertsemlidis A, Cox D, Pennacchio LA, et al Genetic variation in PNPLA3 confers susceptibility to nonalcoholic fatty liver disease. Nat Genet. 2008;40: 1461–1465. 10.1038/ng.257 18820647 PMC2597056

[12] Donati B, Motta BM, Pingitore P, Meroni M, Pietrelli A, Alisi A, et al The rs2294918 E434K variant modulates patatin-like phospholipase domain-containing 3 expression and liver damage. Hepatology. 2016;63: 787–798. 10.1002/hep.28370 26605757

[13] Kozlitina J, Smagris E, Stender S, Nordestgaard BG, Heather H, Tybjærg-hansen A, et al Exome-wide association study identifies a TM6SF2 variant that confers susceptibility to nonalcoholic fatty liver disease. Nat Genet. 2014;46: 352–356. 10.1038/ng.2901 24531328 PMC3969786

[14] Akuta N, Kawamura Y, Arase Y, Suzuki F, Sezaki H, Hosaka T, et al Relationships between Genetic Variations of PNPLA3, TM6SF2 and Histological Features of Nonalcoholic Fatty Liver Disease in Japan. Gut Liver. 2015;10: 1–9. 10.5009/gnl15163 26610348 PMC4849698

[15] Liu Y-L, Reeves HL, Burt AD, Tiniakos D, McPherson S, Leathart JBS, et al TM6SF2 rs58542926 influences hepatic fibrosis progression in patients with non-alcoholic fatty liver disease. Nat Commun. 2014;5 10.1038/ncomms5309 24978903 PMC4279183

[16] Al-Serri A, Anstee QM, Valenti L, Nobili V, Leathart JBS, Dongiovanni P, et al The SOD2 C47T polymorphism influences NAFLD fibrosis severity: Evidence from case-control and intra-familial allele association studies. J Hepatol. 2012;56: 448–454. 10.1016/j.jhep.2011.05.029 21756849

[17] Bale G, Steffie AU, Ravi Kanth VV, Rao PN, Sharma M, Sasikala M, et al Regional differences in genetic susceptibility to nonalcoholic liver disease in two distinct Indian ethnicities. World J Hepatol. 2017;9: 1101–1107. 10.4254/wjh.v9.i26.1101 28989566 PMC5612841

[18] Arendt BM, Comelli EM, Ma DWL, Lou W, Teterina A, Kim T, et al Altered hepatic gene expression in nonalcoholic fatty liver disease is associated with lower hepatic n-3 and n-6 polyunsaturated fatty acids. Hepatology. Wiley-Blackwell; 2015;61: 1565–1578. 10.1002/hep.27695 25581263

[19] Xanthakos SA, Jenkins TM, Kleiner DE, Boyce TW, Mourya R, Karns R, et al High Prevalence of Nonalcoholic Fatty Liver Disease in Adolescents Undergoing Bariatric Surgery. Gastroenterology. 2015;149: 623e8–634e8. 10.1053/j.gastro.2015.05.039 26026390 PMC4654456

[20] Ravi Kanth VV, Sasikala M, Sharma M, Rao PN, Reddy DN. Genetics of non-alcoholic fatty liver disease: From susceptibility and nutrient interactions to management. World J Hepatol. 2016;8: 827–837. 10.4254/wjh.v8.i20.827 27458502 PMC4945502

[21] Yuan X, Waterworth D, Perry JRB, Lim N, Song K, Chambers JC, et al Population-Based Genome-wide Association Studies Reveal Six Loci Influencing Plasma Levels of Liver Enzymes. Am J Hum Genet. 2008;83: 520–528. 10.1016/j.ajhg.2008.09.012 18940312 PMC2561937

[22] Abul-Husn NS, Cheng X, Li AH, Xin Y, Schurmann C, Stevis P, et al A Protein-Truncating HSD17B13 Variant and Protection from Chronic Liver Disease. N Engl J Med. 2018; 10.1056/nejmoa1712191 29562163 PMC6668033

[23] Petersen KF, Dufour S, Hariri A, Nelson-Williams C, Foo JN, Zhang X-M, et al Apolipoprotein C3 gene variants in nonalcoholic fatty liver disease. N Engl J Med. 2010;362: 1082–9. 10.1056/NEJMoa0907295 20335584 PMC2976042

[24] Kitamoto A, Kitamoto T, Nakamura T, Ogawa Y, Yoneda M, Hyogo H, et al Association of polymorphisms in GCKR and TRIB1 with nonalcoholic fatty liver disease and metabolic syndrome traits. Endocr J. 2014;61 10.1507/endocrj.EJ14-0052 24785259

[25] Speliotes EK, Yerges-Armstrong LM, Wu J, Hernaez R, Kim LJ, Palmer CD, et al Genome-wide association analysis identifies variants associated with nonalcoholic fatty liver disease that have distinct effects on metabolic traits. PLoS Genet. 2011;7 10.1371/journal.pgen.1001324 21423719 PMC3053321

[26] Yang Z, Wen J, Tao X, Lu B, Du Y, Wang M, et al Genetic variation in the GCKR gene is associated with non-alcoholic fatty liver disease in Chinese people. Mol Biol Rep. 2011;38: 1145–1150. 10.1007/s11033-010-0212-1 20625834

[27] Petit J-M, Masson D, Guiu B, Rollot F, Duvillard L, Bouillet B, et al GCKR polymorphism influences liver fat content in patients with type 2 diabetes. Acta Diabetol. 2016;53: 237–242. 10.1007/s00592-015-0766-4 25976242

[28] Mancina RM, Dongiovanni P, Petta S, Pingitore P, Meroni M, Rametta R, et al The MBOAT7-TMC4 Variant rs641738 Increases Risk of Nonalcoholic Fatty Liver Disease in Individuals of European Descent. Gastroenterology. 2016;150: 1219–1230e6. 10.1053/j.gastro.2016.01.032 26850495 PMC4844071

[29] Patin E, Kutalik Z, Guergnon J, Bibert S, Nalpas B, Jouanguy E, et al Genome-wide association study identifies variants associated with progression of liver fibrosis from HCV infection. Gastroenterology. 2012;143 10.1186/1471-230X-12-143 22841784 PMC3756935

[30] World Health Organization. Asthma Fact Sheet [Internet]. 2016 [cited 20 Sep 2018]. Available: http://www.who.int/en/news-room/fact-sheets/detail/asthma

[31] Moffatt MF, Gut IG, Demenais F, Strachan DP, Bouzigon E, Heath S, et al A large-scale, consortium-based genomewide association study of asthma. N Engl J Med. 2010;363: 1211–21. 10.1056/NEJMoa0906312 20860503 PMC4260321

[32] Roscioli E, Hamon R, Ruffin RE, Grant J, Hodge S, Zalewski P, et al BIRC3 single nucleotide polymorphism associate with asthma susceptibility and the abundance of eosinophils and neutrophils. J Asthma. 2017;54: 116–124. 10.1080/02770903.2016.1196371 27304223

[33] Astle WJ, Elding H, Jiang T, Allen D, Ruklisa D, Mann AL, et al The Allelic Landscape of Human Blood Cell Trait Variation and Links to Common Complex Disease. Cell. 2016;167: 1415–1429.e19. 10.1016/j.cell.2016.10.042 27863252 PMC5300907

[34] Petta S, Valenti L, Marra F, Grimaudo S, Tripodo C, Bugianesi E, et al MERTK rs4374383 polymorphism affects the severity of fibrosis in non-alcoholic fatty liver disease. J Hepatol. 2016;64: 682–690. 10.1016/j.jhep.2015.10.016 26596542

[35] Melén E, Himes BE, Brehm JM, Boutaoui N, Klanderman BJ, Sylvia JS, et al Analyses of shared genetic factors between asthma and obesity in children. J Allergy Clin Immunol. 2010;126: 631–7.e1-8. 10.1016/j.jaci.2010.06.030 20816195 PMC2941152

[36] Wellen K. E. & Hotamisligil G. S. Inflammation, stress, and diabetes. Journal of Clinical Investigation (2005). 10.1172/JCI200525102 PMC1087185 15864338

[37] Wood KL, Miller MH, Dillon JF. Systematic review of genetic association studies involving histologically confirmed non-Alcoholic fatty liver disease. BMJ Open Gastroenterology. 2015 10.1136/bmjgast-2014-000019 26462272 PMC4599155

[38] Hardy T, Anstee QM, Day CP. Nonalcoholic fatty liver disease: New treatments. Current Opinion in Gastroenterology. 2015 pp. 175–183. 10.1097/MOG.0000000000000175 25774446 PMC4482455

[39] Johnson AD, Handsaker RE, Pulit SL, Nizzari MM, O’Donnell CJ, De Bakker PIW. SNAP: A web-based tool for identification and annotation of proxy SNPs using HapMap. Bioinformatics. 2008;24: 2938–2939. 10.1093/bioinformatics/btn564 18974171 PMC2720775

[40] Neuraz A, Chouchana L, Malamut G, Le Beller C, Roche D, Beaune P, et al Phenome-Wide Association Studies on a Quantitative Trait: Application to TPMT Enzyme Activity and Thiopurine Therapy in Pharmacogenomics. PLoS Comput Biol. 2013;9 10.1371/journal.pcbi.1003405 24385893 PMC3873228

[41] Purcell S, Neale B, Todd-Brown K, Thomas L, Ferreira MAR, Bender D, et al PLINK: A Tool Set for Whole-Genome Association and Population-Based Linkage Analyses. Am J Hum Genet. 2007;81: 559–575. 10.1086/519795 17701901 PMC1950838

[42] Denny JC, Ritchie MD, Basford MA, Pulley JM, Bastarache L, Brown-Gentry K, et al PheWAS: Demonstrating the feasibility of a phenome-wide scan to discover gene-disease associations. Bioinformatics. 2010;26: 1205–1210. 10.1093/bioinformatics/btq126 20335276 PMC2859132

[43] Chambers JC, Zhang W, Sehmi J, Li X, Wass MN, Van Der Harst P, et al Genome-wide association study identifies loci influencing concentrations of liver enzymes in plasma. Nat Genet. 2011;43: 1131–1138. 10.1038/ng.970 22001757 PMC3482372

[44] Verma A, Lucas A, Verma SS, Zhang Y, Josyula N, Khan A, et al PheWAS and Beyond: The Landscape of Associations with Medical Diagnoses and Clinical Measures across 38,662 Individuals from Geisinger. Am J Hum Genet. 2018;102: 592–608. 10.1016/j.ajhg.2018.02.017 29606303 PMC5985339

[45] Verma A, Basile AO, Bradford Y, Kuivaniemi H, Tromp G, Carey D, et al Phenome-Wide association study to explore relationships between immune system related genetic loci and complex traits and diseases. PLoS One. 2016;11 10.1371/journal.pone.0160573 27508393 PMC4980020

[46] Hall MA, Verma A, Brown-Gentry KD, Goodloe R, Boston J, Wilson S, et al Detection of Pleiotropy through a Phenome-Wide Association Study (PheWAS) of Epidemiologic Data as Part of the Environmental Architecture for Genes Linked to Environment (EAGLE) Study. PLoS Genet. 2014;10 10.1371/journal.pgen.1004678 25474351 PMC4256091

[47] Pendergrass SA, Brown-Gentry K, Dudek S, Frase A, Torstenson ES, Goodloe R, et al Phenome-Wide Association Study (PheWAS) for Detection of Pleiotropy within the Population Architecture using Genomics and Epidemiology (PAGE) Network. PLoS Genet. 2013;9 10.1371/journal.pgen.1003087 23382687 PMC3561060

[48] Namjou B, Marsolo K, Caroll RJ, Denny JC, Ritchie MD, Verma SS, et al Phenome-wide association study (PheWAS) in EMR-linked pediatric cohorts, genetically links PLCL1 to speech language development and IL5-IL13 to Eosinophilic Esophagitis. Front Genet. 2014;5 10.3389/fgene.2014.00401 25477900 PMC4235428

[49] Doss J, Mo H, Carroll RJ, Crofford LJ, Denny JC. Phenome-Wide Association Study of Rheumatoid Arthritis Subgroups Identifies Association Between Seronegative Disease and Fibromyalgia. Arthritis Rheumatol. 2017;69: 291–300. 10.1002/art.39851 27589350 PMC5274573

[50] Hebbring SJ, Schrodi SJ, Ye Z, Zhou Z, Page D, Brilliant MH. A PheWAS approach in studying HLA-DRB1*1501. Genes Immun. 2013;14: 187–191. 10.1038/gene.2013.2 23392276 PMC3637423

[51] Verma A, Verma SS, Pendergrass SA, Crawford DC, Crosslin DR, Kuivaniemi H, et al EMERGE Phenome-Wide Association Study (PheWAS) identifies clinical associations and pleiotropy for stop-gain variants. BMC Med Genomics. 2016;9 10.1186/s12920-016-0191-8 27535653 PMC4989894

[52] Hebbring SJ, Rastegar-Mojarad M, Ye Z, Mayer J, Jacobson C, Lin S. Application of clinical text data for phenome-wide association studies (PheWASs). Bioinformatics. 2015;31: 1981–1987. 10.1093/bioinformatics/btv076 25657332 PMC4481696

[53] Kozlitina J, Boerwinkle E, Cohen JC, Hobbs HH. Dissociation between APOC3 variants, hepatic triglyceride content and insulin resistance. Hepatology. 2011;53: 467–474. 10.1002/hep.24072 21274868 PMC3057507

[54] Van Kaer L. Major histocompatibility complex class I-restricted antigen processing and presentation. Tissue Antigens. 2002 pp. 1–9. 10.1034/j.1399-0039.2002.600101.x 12366777

[55] Ramgopal S, Rathika C, Padma Malini R, Murali V, Arun K, Balakrishnan K. Critical amino acid variations in HLA-DQB1* molecules confers susceptibility to Autoimmune Thyroid Disease in south India. Genes Immun. Nature Publishing Group; 2018; 1 10.1038/s41435-017-0008-6 29307887

[56] Engelbrecht Zantut-Wittmann D, Persoli L, Tambascia MA, Fischer E, Franco Maldonado D, Costa AM, et al HLA-DRB1*04 and HLA-DQB1*03 association with the atrophic but not with the goitrous form of chronic autoimmune thyroiditis in a Brazilian population. Horm Metab Res. 2004;36: 492–500. 10.1055/s-2004-825732 15305234

[57] Giza S, Galli-Tsinopoulou A, Lazidou P, Trachana M, Goulis D. HLA-DQB1*05 association with Hashimoto’s thyroiditis in children of Northern Greek origin. Indian Pediatr. 2008;45: 493–6. 10.1542/peds.2007-2022JJ 18599937

[58] Santamaria P, Barbosa JJ, Lindstrom AL, Lemke TA, Goetz FC, Rich SS. HLA-DQB1-associated susceptibility that distinguishes Hashimoto’s thyroiditis from Graves’ disease in type I diabetic patients. J Clin Endocrinol Metab. 1994;78: 878–83. 10.1210/jcem.78.4.8157715 8157715

[59] Mosaad YM, Auf FA, Metwally SS, Elsharkawy AA, El-Hawary AK, Hassan RH, et al HLA-DQB1* alleles and genetic susceptibility to type 1 diabetes mellitus. World J Diabetes. 2012;3: 149–55. 10.4239/wjd.v3.i8.149 22919445 PMC3425629

[60] Gao J, Lin Y, Qiu C, Liu Y, Ma Y, Liu Y. Association between HLA-DQA1, -DQB1 gene polymorphisms and susceptibility to asthma in northern Chinese subjects. Chin Med J (Engl). 2003;116: 1078–1082. 12890388

[61] Movahedi M, Moin M, Gharagozlou M, Aghamohammadi A, Dianat S, Moradi B, et al Association of HLA class II alleles with childhood asthma and total IgE levels. Iran J Allergy, Asthma Immunol. 2008;7: 215–220. Available: http://www.embase.com/search/results?subaction=viewrecord&from=export&id=L352801461%5Cnhttp://www.iaari.hbi.ir/journal/archive/articles/v7n4ami.pdf%5Cnhttp://vb3lk7eb4t.search.serialssolutions.com?sid=EMBASE&issn=17351502&id=doi:&atitle=Association+of+HLA 19052351

