# Supplementary material for: An exploratory phenome wide association study linking asthma and liver disease genetic variants to electronic health records from the Estonian Biobank
Source: PLoS One. 2019 Apr 12;14(4):e0215026. doi: 10.1371/journal.pone.0215026 (PMC6461350; doi:10.1371/journal.pone.0215026)

S1 Text. Summary statistics and distributions for each laboratory/biomarker measurements.

Table A - Summary statistics for each laboratory / biomarker measurement. Measurements where the logarithm was taken before an analysis are marked with a star (*)

| **Measure** | **N_total** | **N_patients** | **Mean** | **SD** | **Min** | **1st quartile** | **Median** | **3rd quartile** | **Max** |
| --- | --- | --- | --- | --- | --- | --- | --- | --- | --- |
| WBC* | 59936 | 12859 | 1.952976 | 0.485283 | -4.60517 | 1.704748 | 1.94591 | 2.203869 | 5.388752 |
| CRP* | 54805 | 10928 | 2.278205 | 1.735029 | -4.60517 | 0.850151 | 2.116256 | 3.688879 | 6.556153 |
| CREATININE* | 50400 | 9702 | 4.537723 | 0.649127 | 0.55727 | 4.158883 | 4.356709 | 4.662213 | 8.437284 |
| ALT* | 34133 | 9107 | 3.114 | 0.775651 | 0 | 2.639057 | 2.995732 | 3.465736 | 8.52397 |
| HAEMO | 44767 | 8970 | 123.9494 | 20.84509 | 0 | 110 | 125 | 139 | 214 |
| POTASSUM | 53899 | 8815 | 4.325406 | 0.61656 | 0.8 | 3.9 | 4.3 | 4.6 | 17.4 |
| SODIUM | 50548 | 8806 | 138.8433 | 3.906957 | 95 | 137 | 139 | 141 | 175 |
| UREA* | 40038 | 8588 | 1.901407 | 0.606406 | -1.20397 | 1.504077 | 1.808289 | 2.197225 | 6.408529 |
| NEUTRO* | 39070 | 8174 | 1.356417 | 0.78845 | -4.60517 | 1.033184 | 1.378766 | 1.733424 | 4.53903 |
| EOSIN* | 39700 | 8040 | -2.16413 | 1.325745 | -4.60517 | -2.81341 | -2.12026 | -1.46968 | 3.504957 |
| PLATELET | 40026 | 7636 | 240.1912 | 95.44511 | 0 | 184 | 230 | 282 | 1254 |
| AST* | 28314 | 7242 | 3.212509 | 0.630906 | 1.386294 | 2.833213 | 3.091042 | 3.401197 | 9.462188 |
| CHOLLEV | 13679 | 5887 | 5.407714 | 1.419289 | 1.3 | 4.5 | 5.3 | 6.2 | 23.9 |
| BILRUBIN* | 17301 | 4996 | 2.271089 | 0.708227 | 0 | 1.791759 | 2.197225 | 2.639057 | 6.44254 |
| LDLCHOL | 9212 | 4394 | 3.401133 | 1.249424 | 0.11 | 2.59 | 3.29 | 4.08 | 17.38 |
| HDLCHOL | 9187 | 4393 | 1.389935 | 0.46588 | 0.1 | 1.06 | 1.33 | 1.64 | 4.63 |
| FASTGLUC* | 10358 | 4284 | 1.880241 | 0.398202 | -1.23787 | 1.629241 | 1.774952 | 2.04122 | 4.168214 |
| GGT* | 9218 | 4179 | 3.627318 | 1.109136 | 1.386294 | 2.772589 | 3.367296 | 4.204693 | 8.051022 |
| ALP | 10680 | 3887 | 4.446831 | 0.560985 | 2.772589 | 4.110874 | 4.343805 | 4.656099 | 8.402231 |
| SERUMUA | 9810 | 3226 | 350.4205 | 116.9157 | 51 | 269.0808 | 336 | 416 | 1107 |
| ALBUMIN | 9871 | 2805 | 38.48012 | 7.258942 | 0.2 | 34 | 40 | 44 | 59 |
| NTBNP* | 3426 | 1713 | 6.158303 | 1.762961 | 1.609438 | 4.844187 | 6.012485 | 7.410044 | 11.20421 |
| TROPT* | 1713 | 644 | -1.95234 | 2.129206 | -9.36116 | -3.7297 | -2.3969 | -0.15082 | 3.41871 |
| FIBRINOGEN | 247 | 185 | 4.212146 | 2.747673 | 1.2 | 2.685 | 3.5 | 5 | 29.7 |
| AFETO* | 127 | 120 | 0.857023 | 0.64502 | -0.69315 | 0.388376 | 0.850151 | 1.308318 | 2.761275 |
| TROPI* | 208 | 113 | -3.78895 | 1.358024 | -6.90776 | -4.60517 | -3.91202 | -3.21888 | 0.253867 |

Figure A - distributions of all biomarker / lab measures


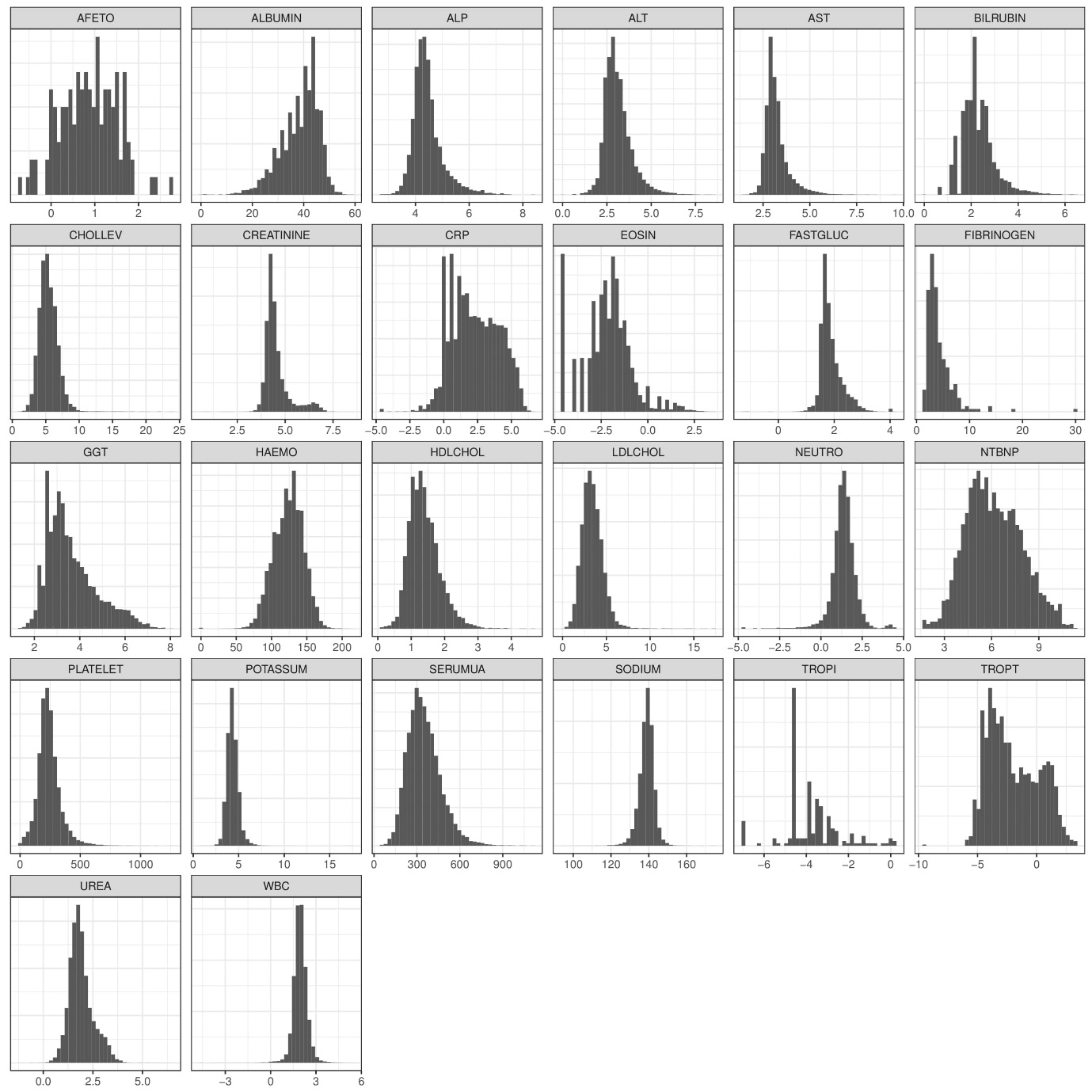

Supplement: S1 Text — (DOCX) [file pone.0215026.s002.docx]
