# Supplementary material for: An exploratory phenome wide association study linking asthma and liver disease genetic variants to electronic health records from the Estonian Biobank
Source: PLoS One. 2019 Apr 12;14(4):e0215026. doi: 10.1371/journal.pone.0215026 (PMC6461350; doi:10.1371/journal.pone.0215026)
Supplement: S2 Text — (DOCX) [file pone.0215026.s003.docx]

**S2 Text. Calculations for statistical power**

The statistical power to detect an association between case-control status and a genetic variant is shown in Figure A. For example, specifying a significance level (alpha) of 0.0500, a two-sided Z test with pooled variance and group sample sizes of 50,000 in group one and 600 in group two achieves 96% power to detect a ratio in the group proportions of 1.500. The proportion in group one (the treatment group) is assumed to be 0.1000 under the null hypothesis and 0.1500 under the alternative hypothesis. The proportion in group two (the control group) is 0.1000.

Figure A - The statistical power to detect and association between case-control status and a genetic variant. N1=sample size in case group (50,000); N2=sample size in control group; P1=proportion with variant in case group; P2=proportion with variant in control group; R1=P1/P2 on alternative hypothesis (H1) (1.50); H0=P1/P2=1; A=probability of rejecting H0 if it is true (2-sided Z-test)

The statistical power of the proposed study to detect association between a quantitative trait and a genetic variant is shown in Figure B. For example, assuming a coefficient of variation (CV) of 0.150 and specifying a significance level (alpha) of 0.0500, a two-sided, two-sample t-test with group sample sizes of 50,000 and 600 achieves 99% power to detect a ratio of 0.975 when the ratio under the null hypothesis is 1.000.

Figure B - The statistical power of the proposed study to detect association between a quantitative trait and a genetic variant. N1=sample size in case group (50,000); N2=sample size in control group; R0=Mean2/Mean1 under the null hypothesis (H0) (1.00); R1=Mean2/Mean1 at which the power is calculated; CV=coefficient of variation on the original scale (0.15); Alpha= probability of rejecting H0 if it is true (2-sided t-test)

**
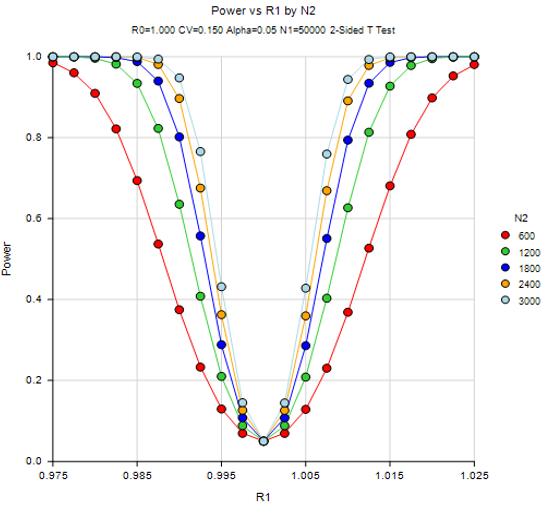
**
