# Supplementary material for: An exploratory phenome wide association study linking asthma and liver disease genetic variants to electronic health records from the Estonian Biobank
Source: PLoS One. 2019 Apr 12;14(4):e0215026. doi: 10.1371/journal.pone.0215026 (PMC6461350; doi:10.1371/journal.pone.0215026)
Supplement: S3 Text — (DOCX) [file pone.0215026.s004.docx]

**S3 Text. Supplementary figures**


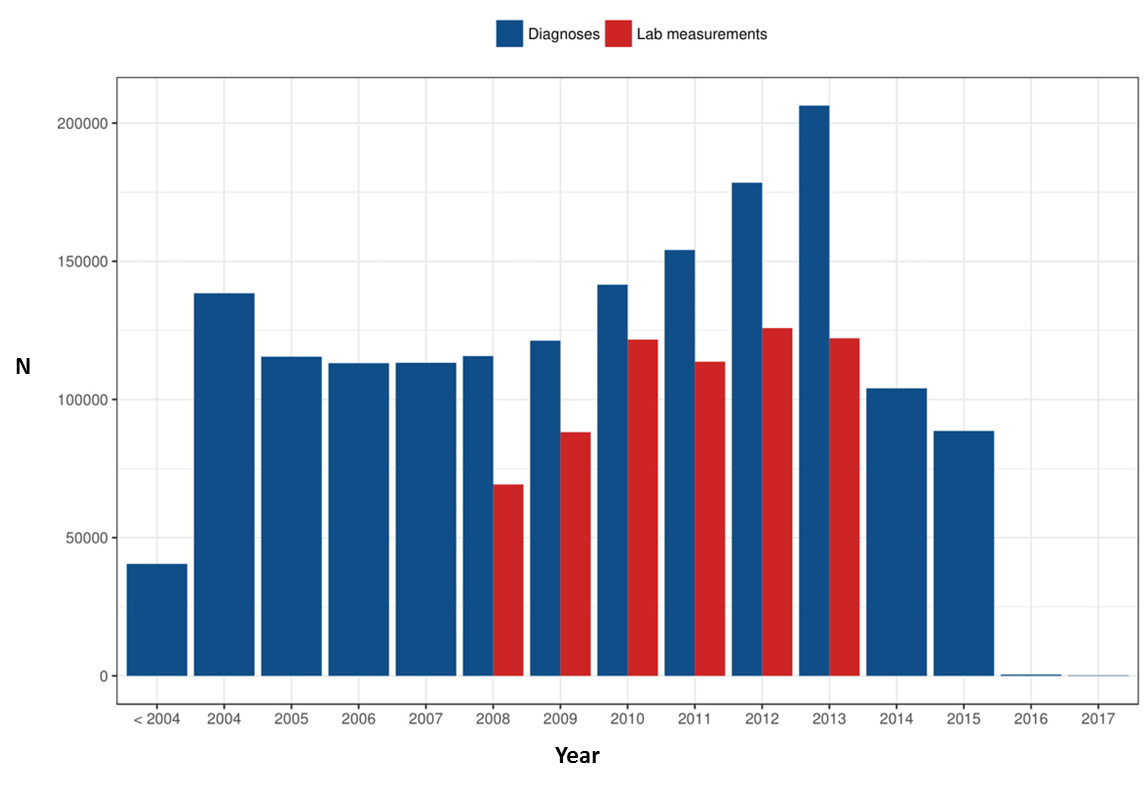


Figure A - Number of disease diagnoses and laboratory/biomarker measurements by year in the Estonian Biobank dataset.


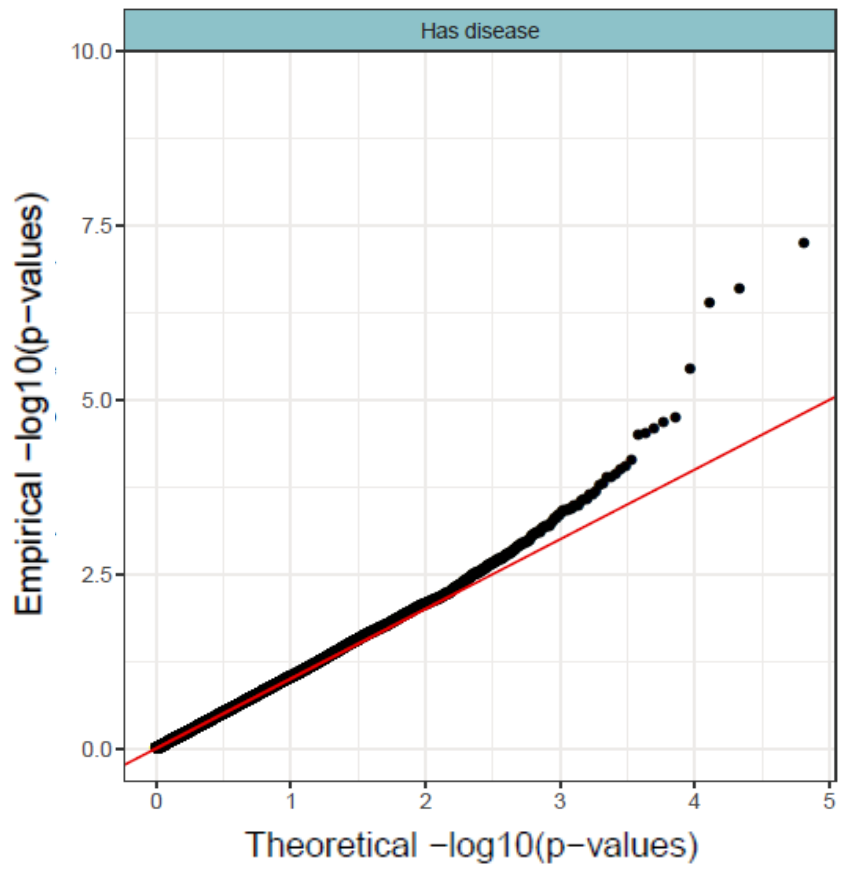


Figure B - QQ-Plot for PheWAS p-value distribution
