# Supplementary material for: An exploratory phenome wide association study linking asthma and liver disease genetic variants to electronic health records from the Estonian Biobank
Source: PLoS One. 2019 Apr 12;14(4):e0215026. doi: 10.1371/journal.pone.0215026 (PMC6461350; doi:10.1371/journal.pone.0215026)
Supplement: S4 Text — (DOCX) [file pone.0215026.s005.docx]

**S4 Text. Liver Disease and Asthma ICD-10 Diagnostic Codes with occurrence and patient count**

### Table A. Asthma Specific Codes:

| **ICD-10 code** | **ICD-10 name** | **Number of occurrences** | **Number of patients** |
| --- | --- | --- | --- |
| J45 | Asthma | 1885 | 1620 |
| J45.0 | Predominantly allergic asthma | 1156 | 814 |
| J45.1 | Nonallergic asthma | 849 | 473 |
| J45.8 | Mixed Asthma | 4170 | 1954 |
| J45.9 | Asthma, Unspecified | 2220 | 1523 |
| J46 | Status asthmaticus | 47 | 42 |

### Table B. Codes in range J40-J47 used as an exclusion for asthma controls:

| **ICD-10 code** | **ICD-10 name** | **Number of occurrences** | **Number of patients** |
| --- | --- | --- | --- |
| J40 | Bronchitis, not specified as acute or chronic | 1225 | 1084 |
| J41 | Simple and mucopurulent chronic bronchitis | 503 | 471 |
| J41.0 | Simple chronic bronchitis | 830 | 717 |
| J41.1 | Mucopurulent chronic bronchitis | 216 | 189 |
| J41.8 | Mixed simple and mucopurulent chronic bronchitis | 398 | 332 |
| J42 | Unspecified chronic bronchitis | 1036 | 923 |
| J43 | Emphysema | 58 | 53 |
| J43.0 | Unilateral pulmonary emphysema [MacLeod's syndrome] | 4 | 4 |
| J43.1 | Panlobular emphysema | 22 | 14 |
| J43.2 | Centrilobular emphysema | 48 | 27 |
| J43.8 | Other emphysema | 70 | 50 |
| J43.9 | Emphysema, unspecified | 58 | 48 |
| J44 | Other chronic obstructive pulmonary disease | 692 | 626 |
| J44.0 | Chronic obstructive pulmonary disease with acute lower respiratory infection | 426 | 273 |
| J44.1 | Chronic obstructive pulmonary disease with (acute) exacerbation | 288 | 206 |
| J44.8 | Other specified chronic obstructive pulmonary disease | 1914 | 962 |
| J44.9 | Chronic obstructive pulmonary disease, unspecified | 801 | 553 |
| J45 | Asthma | 1885 | 1620 |
| J45.0 | Predominantly allergic asthma | 1156 | 814 |
| J45.1 | Nonallergic asthma | 849 | 473 |
| J45.8 | Mixed Asthma | 4170 | 1954 |
| J45.9 | Asthma, Unspecified | 2220 | 1523 |
| J46 | Status asthmaticus | 47 | 42 |
| J47 | Bronchiectasis | 403 | 156 |

### Table C. Liver Specific Codes:

| **ICD-10 code** | **ICD-10 name** | **Number of occurrences** | **Number of patients** |
| --- | --- | --- | --- |
| K70 | Alcoholic liver disease | 55 | 49 |
| K70.0 | Alcoholic fatty liver | 133 | 95 |
| K70.1 | Alcoholic hepatitis | 113 | 87 |
| K70.2 | Alcoholic fibrosis and sclerosis of liver | 9 | 6 |
| K70.3 | Alcoholic cirrhosis of the liver | 153 | 69 |
| K70.4 | Alcoholic hepatic failure | 12 | 12 |
| K70.9 | Alcoholic liver disease, unspecified | 53 | 42 |
| K71 | Toxic liver disease | 12 | 12 |
| K71.0 | Toxic liver disease with cholestasis | 23 | 19 |
| K71.1 | Toxic liver disease with hepatic necrosis | 4 | 3 |
| K71.2 | Toxic liver disease with acute hepatitis | 6 | 5 |
| K71.3 | Toxic liver disease with chronic persistent hepatitis | 0 | 0 |
| K71.4 | Toxic liver disease with chronic lobular hepatitis | 0 | 0 |
| K71.5 | Toxic liver disease with chronic active hepatitis | 1 | 1 |
| K71.6 | Toxic liver disease with hepatitis, not elsewhere classified | 1 | 1 |
| K71.7 | Toxic liver disease with fibrosis and cirrhosis of liver | 2 | 1 |
| K71.8 | Toxic liver disease with other disorders of liver | 5 | 5 |
| K71.9 | Toxic liver disease, unspecified | 20 | 18 |
| K72 | Hepatic failure, not elsewhere classified | 16 | 16 |
| K72.0 | Acute and subacute hepatic failure | 16 | 13 |
| K72.1 | Chronic hepatic failure | 15 | 12 |
| K72.9 | Hepatic failure, unspecified | 24 | 18 |
| K73 | Chronic hepatitis, not elsewhere classified | 130 | 125 |
| K73.0 | Chronic persistent hepatitis, not elsewhere classified | 29 | 29 |
| K73.1 | Chronic lobular hepatitis, not elsewhere classified | 1 | 1 |
| K73.2 | Chronic active hepatitis, not elsewhere classified | 52 | 25 |
| K73.8 | Other chronic hepatitis, not elsewhere classified | 70 | 50 |
| K73.9 | Chronic hepatitis, unspecified | 236 | 201 |
| K74 | Fibrosis and cirrhosis of liver | 29 | 28 |
| K74.0 | Hepatic fibrosis | 22 | 9 |
| K74.1 | Hepatic sclerosis | 1 | 1 |
| K74.2 | Hepatic fibrosis with hepatic sclerosis | 4 | 3 |
| K74.3 | Primary biliary cirrhosis | 175 | 36 |
| K74.4 | Secondary biliary cirrhosis | 4 | 4 |
| K74.5 | Biliary cirrhosis, unspecified | 4 | 4 |
| K74.6 | Other and unspecified cirrhosis of liver | 130 | 64 |
| K75 | Other inflammatory liver diseases | 59 | 59 |
| K75.0 | Abscess of liver | 22 | 12 |
| K75.1 | Phlebitis of portal vein | 0 | 0 |
| K75.2 | Nonspecific reactive hepatitis | 10 | 8 |
| K75.3 | Granulomatous hepatitis, not elsewhere classified | 3 | 2 |
| K75.4 | Autoimmune hepatitis | 0 | 0 |
| K75.8 | Other specified inflammatory liver diseases, Nonalcoholic steatohepatitis (NASH) | 35 | 26 |
| K75.9 | Inflammatory liver disease, unspecified | 128 | 109 |
| K76 | Other diseases of liver, Non-alcoholic fatty liver disease (NAFLD) | 177 | 168 |
| K76.0 | Fatty (change of) liver, not elsewhere classified | 828 | 605 |
| K76.1 | Chronic passive congestion of liver | 20 | 12 |
| K76.2 | Central haemorrhagic necrosis of liver | 1 | 1 |
| K76.3 | Infarction of liver | 1 | 1 |
| K76.4 | Peliosis hepatis | 7 | 6 |
| K76.5 | Hepatic veno-occlusive disease | 2 | 2 |
| K76.6 | Portal hypertension | 108 | 47 |
| K76.7 | Hepatorenal syndrome | 20 | 15 |
| K76.8 | Other specified diseases of liver | 167 | 133 |
| K76.9 | Liver disease, unspecified | 268 | 221 |
| K77 | Liver disorders in diseases classified elsewhere | 2 | 2 |
| K77.0 | Liver disorders in infectious and parasitic diseases classified elsewhere | 4 | 4 |
| K77.8 | Liver disorders in other diseases classified elsewhere | 8 | 8 |
